# Transcriptomic Signatures Associated With Gray Matter Volume Changes in Patients With Functional Constipation

**DOI:** 10.3389/fnins.2021.791831

**Published:** 2022-01-05

**Authors:** Wangli Cai, Yujing Zhou, Lidi Wan, Ruiling Zhang, Ting Hua, Jian Gong, Bo Yang, Guangyu Tang

**Affiliations:** ^1^Department of Radiology, Shanghai Tenth People’s Hospital, School of Clinical Medicine of Nanjing Medical University, Shanghai, China; ^2^Department of Radiology, The First Affiliated Hospital of Dalian Medical University, Dalian, China; ^3^Department of Colorectal and Anal Surgery, Shanghai Tenth People’s Hospital, School of Clinical Medicine of Nanjing Medical University, Shanghai, China

**Keywords:** functional constipation, Allen Human Brain Atlas, gene expression analysis, gray matter volume, imaging-genetics

## Abstract

Functional constipation, which belongs to the functional gastrointestinal disorder (FGID), is a common disease and significantly impacts daily life. FGID patients have been progressively proven with functional and structural alterations in various brain regions, but whether and how functional constipation affects the brain gray matter volume (GMV) remains unclear; besides, which genes are associated with the GMV changes in functional constipation is largely unknown. On account of the structural MRI image from the 30 functional constipation patients and 30 healthy controls (HCs), GMV analysis showed that functional constipation patients had significantly decreased GMV in the right orbital prefrontal cortex (OFC), left precentral gyrus (PreG), and bilateral thalamus (THA). Correlation analysis showed that the self-rating depressive scale, patient assessment of constipation quality of life (PAC-QOL), and Wexner constipation scores were negatively correlated with GMV of the OFC and negative correlations between PAC-QOL score and GMV of the bilateral THA. Based on the Allen Human Brain Atlas, a cross-sample spatial correlation was conducted and found that 18 genes’ expression values showed robust correlations with GMV changes in functional constipation patients. These outcomes highlight our recognition of the transcriptional features related to GMV changes in functional constipation and could be regarded as candidates to detect biological mechanisms of abnormality in functional constipation patients.

## Introduction

Functional constipation, as one type of the functional gastrointestinal disorder (FGID), is portrayed by rare bowel movements, painful defecation, uncomfortable feeling of incomplete evacuation, hard/big stools, and frequently joined with abdominal distension and/or abdominal pain (Alame and Bahna, 2012). The incidence rate of functional constipation is relatively high in the entire community, about 0.7–79% (Mugie et al., 2011). Besides, an impressive proportion of functional constipation patients would accompany anxiety and varying severity of depression (Hosseinzadeh et al., 2011). These symptoms severely sway the quality of their daily life and emotional status (Glia and Lindberg, 1997; Bongers et al., 2009).

At present, robust evidence demonstrated that FGID results from physiological changes of the gastrointestinal system because of the bidirectional brain–gut axis, which may influence brain functional/structural alterations, and subsequently arising from anxiety and depression symptoms (Mayer et al., 2006; Mazur et al., 2012). Accordingly, neuroimaging methods have been progressively applied to investigate brain structural and functional anomalies of FGID patients (Blankstein et al., 2010; Zhu et al., 2016; Jin et al., 2019; Hu et al., 2020; Duan et al., 2021; Liu et al., 2021). They found the functional abnormalities in brain regions enrolled in emotion modulating, including the orbitofrontal cortex (OFC), anterior insula, dorsal anterior cingulate cortex, and hippocampus, and motor control, including the precentral gyrus and supplementary motor area. Besides, several pieces of research in FGID patients demonstrated functional impairments in the thalamus, assuming a fundamental part in sensory and motor signal processing (Tillisch et al., 2011; Liu et al., 2021). Moreover, a new structural MRI research had shown that functional constipation patients demonstrated significant diminished cortical thickness of different brain regions, including the left orbitofrontal cortex, left middle frontal gyrus, left medial prefrontal gyrus, left supplementary motor area, right dorsal anterior cingulate cortex, right middle temporal gyrus, and bilateral posterior cingulate cortex/precuneus; decreased cortical volume in the left middle temporal gyrus and bilateral posterior cingulate cortex/precuneus; and induced cortical surface area in the right precentral gyrus additionally (Hu et al., 2020). The above-mentioned brain regions are primarily associated with somatic movement controlling and emotion modulating (Zhu et al., 2016; Hu et al., 2020), and these alterations were implicated in the symptoms of functional constipation patients containing disorder of defecation and unhealthy mood (Ringel, 2002; Alame and Bahna, 2012; Al Omran and Aziz, 2014). Notwithstanding, there has been no study of transcriptional neuroimaging analysis to identify genes related to GMV alterations in functional constipation patients.

**FIGURE 1:**
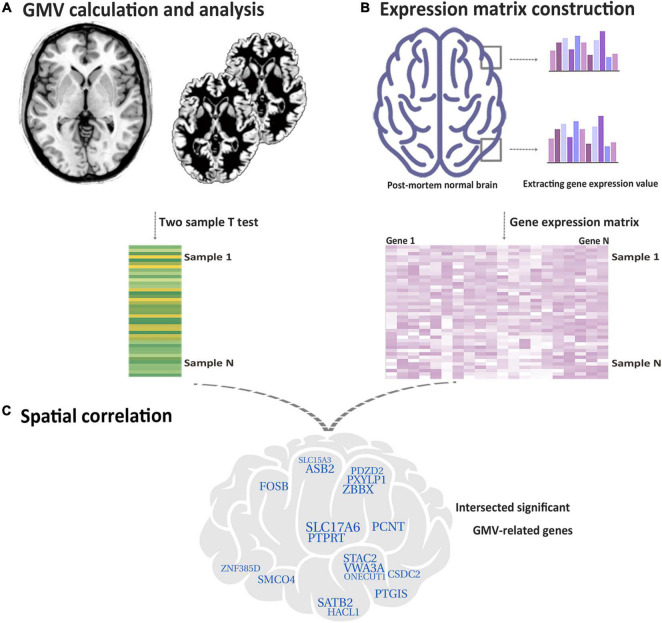
Flow chart of the study design. **(A)** Based on functional constipation and HC, utilizing the two-sample *t*-test to make voxel-wise GMV changes for each tissue sample. **(B)** Acquired gene expression values in each tissue sample. **(C)** Identifying genes related with GMV changes in functional constipation. The crossed specific genes are characterized as genes related with GMV changes in functional constipation.

Currently, based on the Allen Human Brain Atlas (AHBA)^1^, we acquired the gene expression data and conducted the transcriptional neuroimaging analysis to find the genes related to GMV changes in functional constipation patients. We extracted gene expression data from each sample and calculated GMV changes depending on the high-resolution structural MRI image of functional constipation and controls, in sequence, cross-sample spatial association analysis between GMV changes and gene expression values. An illustration of the handling flow chart is displayed in Figure 1.

## Materials and Methods

### Subjects

The experiment was approved by the Ethical Committee of Shanghai Tenth People’s Hospital, and each subject gave written informed consent before the study. Utilizing Rome IV criteria (Drossman, 2016), patients diagnosed by a gastroenterologist expert as having functional constipation with history over 1 year were enrolled in our study. Subjects were barred from the study if they had the following diseases, namely, congenital giant colon, excess sigmoid colon, and mental disease, or who were drug abusers. Besides, subjects with other brain disorders or abnormalities (such as severe white matter hyperintensity, lacunes, microbleeds, and tumors), as determined using T2 fluid-attenuated inversion recovery (FLAIR) sequence, were excluded. Finally, there were 30 right-handed patients with functional constipation (10 men, right-handed, 46.00 ± 18.03 years) who finished the MRI scans. The healthy control group comprised 30 subjects who were right-handed and age and gender matched (9 men, right-handed, 45.77 ± 14.63 years). Self-directed surveys, including the patient assessment of constipation quality of life (PAC-QOL) and Wexner constipation score, were displayed to all members to comprehensively assess the burden of constipation on patients’ regular working and life (Marquis et al., 2005). Patients were additionally approached to finish the Zung (1965) self-rating depressive scale (SDS) and Zung (1971) self-rating anxiety scale (SAS) to survey their seriousness of depression and anxiety.

### Data Acquisition and Gray Matter Volume Calculation

Sagittal 3D high-resolution T1-weighted data were collected by a turbo field echo (TFE) sequence. The parameters are as followed: repetition time (TR)/echo time (TE) = 7.0 ms/3.2 ms; field of view = 256 mm × 256 mm; matrix = 256 × 256. The thickness slice is 1.0 mm, and there were 192 slices with no gap (Ingenia 3.0, Philips).

All the structural MRI data were preprocessed utilizing CAT12 software (version r1364) with the accompanying methodology: bias correction, segmentation, the creation of population-specific tissue templates, spatial normalization using the DARTEL technique, and smoothing with an 8 mm × 8 mm × 8 mm full-width. After these preprocessing steps, we acquired the normalized, modulated, and smoothed GMV images, and each voxel represented volume information.

### Case–Control Gray Matter Volume Changes

We conducted the voxel-based comparisons to distinguish the brain regions that showed group differences in GMV by utilizing the two-sample *t*-test, controlling the impacts of gender, age, and whole intracranial volume. The multiple comparisons were adjusted by the false discovery rate method (*p* < 0.05) and a cluster size >200 voxels using the SPM12 software. Then, a region of interest (ROI)-based association analysis was applied to test relationships between the GMVs of the brain areas, which showed significant group differences with the SDS, SAS, PAC-QOL, and Wexner constipation scores.

### Gene Expression Data Preprocessing

Freely accessible gene expression data of six postmortem human brains were obtained from the AHBA dataset (Hawrylycz et al., 2012), and they were handled utilizing a new flow chart to combine gene expression data with neuroimage data (Arnatkeviciute et al., 2019). We divided the AHBA dataset into two datasets: the first dataset consisted of two donors with the whole brain gene expression data, which have 820 samples, and the second dataset consisted of six donors only with the left-brain gene expression data, which have 1,782 samples. The short processing work was as follows: first, we reassigned probes to genes by using the most recent sequencing databases; second, we barred probes with lower expression signal intensity compared to background noise; third, the probes showing high consistency with the RNA-sequence data were picked; lastly, the expression data were normalized. After such handling process, we obtained 10,185 genes with a normalized expression value of each sample.

### Genes Associated With Gray Matter Volume Changes in Functional Constipation Patients

After extracting gene expression data from each sample (820/1,782 samples) and calculating GMV changes (t-statistic values) in these samples derived from the two-sample *t*-test based on the high-resolution structural MRI image of functional constipation patients and HC, we performed a gene-wise cross-sample Spearman correlation analysis to decide the relationships between GMV changes and gene expression values independently (*n* = 10,185). Considering the multiple comparisons at the gene level (*n* = 10,185), we adjusted the Bonferroni correction method and set a *p* < 4.91 × 10^–7^ = 0.05/10,185 to identify the significant genes. At last, the genes related to GMV changes in functional constipation patients were characterized as those whose expression values derived from two expression datasets were prominently associated with GMV changes.

## Results

### Demographic and Clinical Characteristics

The general clinical characteristics of functional constipation patients and HC are summed up in Table 1. The two groups have not shown significant differences in gender (χ^2^ = 0.077, df = 1, *p* = 0.781), age (*F* = 2.451, df = 58, *p* = 0.123), body mass index (BMI) (*F* = 0.287, df = 58, *p* = 0.594), and anxiety (*F* = 1.582, df = 58, *p* = 0.213) between the two groups. There was significant group difference on depression (*F* = 7.474, df = 58, *p* = 0.008).

**TABLE 1:** The general clinical characteristics of functional constipation and HC.

	FCon (*N* = 30) (Mean ± SE)	HC (*N* = 30) (Mean ± SE)	FCon vs. HC *p*-value
Age (years)	46.00 ± 18.03	45.77 ± 14.63	0.123
Gender	10M/20F	9M/21F	0.781
BMI (kg/m^2^)	22.62 ± 3.17	21.53 ± 2.87	0.594
Depression (SDS)	53.47 ± 10.74	30.93 ± 7.01	0.008
Anxiety (SAS)	50.60 ± 9.76	33.27 ± 7.34	0.213
PAC-QOL	57.27 ± 20.18	N/A	N/A
Wexner constipation score	13.47 ± 3.35	N/A	N/A

*FCon, patients with functional constipation; HC, healthy controls; SE, standard error; BMI, body mass index; SDS, Zung Self-rating Depressive Scale; SAS, Zung Self-Rating Anxiety Scale; PAC-QOL, patient assessment of constipation quality of life.*

### Gray Matter Volume Changes of Functional Constipation Patients

**FIGURE 2:**
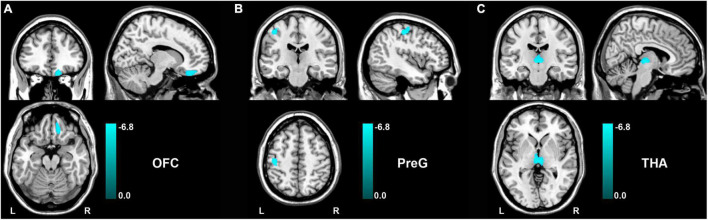
GMV changes between functional constipation and healthy controls (*p* < 0.05, FDR correction). The color bar showed the range of *t*-value. There was significantly decreased GMV in the right OFC **(A)**, left PreG **(B)**, and bilateral THA **(C)**. GMV, gray matter volume; L, left; OFC, orbital prefrontal cortex; PreG, precentral gyrus; R, right; THA, thalamus.

After analyzing voxel-wise GMV alterations (*P* < 0.05, FDR corrected) between the patients with functional constipation and HC, compared to HC groups, we found that functional constipation patients primarily demonstrated decreased GMV in the right orbital prefrontal cortex (OFC, peak MNI coordinates: *x* = 15, *y* = 37.5, *z* = −22.5; cluster size = 230 voxels), left precentral gyrus (PreG, peak MNI coordinates: *x* = −42, *y* = 24, *z* = 52.5; cluster size = 230 voxels), and bilateral thalamus (THA, peak MNI coordinates: *x* = 4.5, *y* = −19.5, *z* = −1.5; cluster size = 338 voxels) (Figure 2). In addition, there were no regions demonstrating a significant increased GMV in functional constipation patients.

**FIGURE 3:**
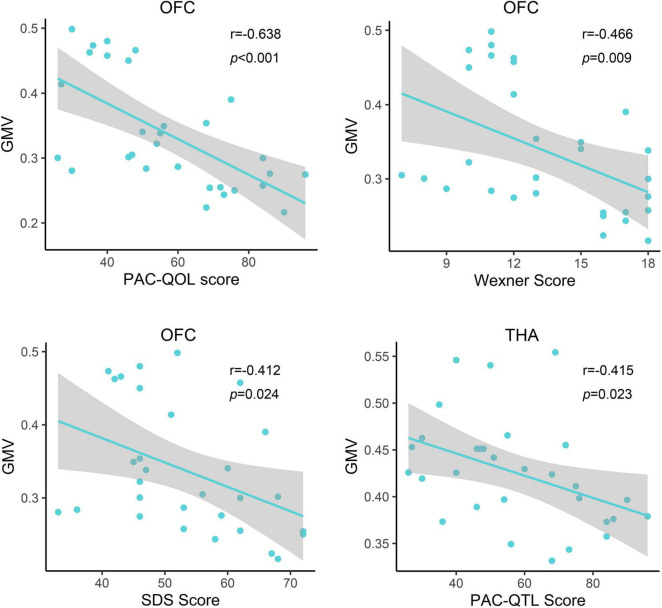
Relationship between clinical information and gray matter volume of the impaired brain region in functional constipation patients. In the functional constipation patients, the PAC-QOL, Wexner constipation score, and SDS score were negatively correlated with GMV of the right OFC. There was negative correlation between PAC-QOL score and GMV of the bilateral THA. GMV, gray matter volume; L, left; OFC, orbital prefrontal cortex; PAC-QOL, patient assessment of constipation quality of life; PreG, precentral gyrus; R, right; SDS, self-rating depressive scale; THA, thalamus.

Correlation analysis showed that the PAC-QOL, Wexner constipation score, and SDS score were negatively correlated with GMV of the OFC (*r* = −0.638, *p* < 0.001; *r* = −0.466, *p* = 0.009; and *r* = −0.412, *p* = 0.024, respectively) in functional constipation patients (Figure 3). There was negative correlation between PAC-QOL score and GMV of the bilateral THA (*r* = −0.415, *p* = 0.023) in functional constipation patients (Figure 3). However, the data showed expected heterogeneity due to the small sample size; we should restrict the wide application of such conclusions, as there exist correlated tendencies, which need further verification in the future.

### Genes Related to Gray Matter Volume Changes in Functional Constipation Patients

**FIGURE 4:**
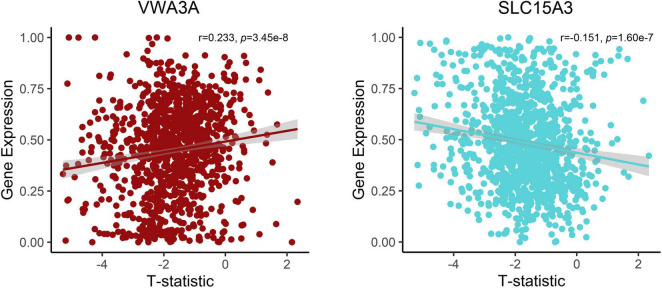
Correlations between expression of two representative genes and GMV changes in patients with functional constipation. The *x*-axis shows the T-statistic of GMV difference between functional constipation patients and healthy controls, and the *y*-axis is the gene expression value.

After the gene expression data processing, we ultimately achieved 10,185 genes with normalized expression values for every 820 and 1,782 samples from the two AHBA datasets. A cross-sample spatial correlation was conducted between GMV changes in functional constipation patients and gene expression value. There were 345 genes that revealed a significant correlation with GMV changes in functional constipation in the first dataset and 208 genes in the second (*p* < 0.05, Bonferroni corrected). The crossed 18 genes of the two AHBA expression datasets were chosen. Detailed descriptions and correlation coefficients of these genes are exhibited in Table 2. Hence, the positive correlation that implies higher gene expression in brain samples manifested a more prominent GMV decrease in functional constipation patients. The negative correlation that implies lower gene expression in brain samples manifested a more noteworthy GMV decrease in functional constipation patients (Figure 4).

**TABLE 2:** The candidate 18 genes manifesting prominent relationships between gene expression and GMV alterations in functional constipation.

Gene symbol	Correlation coefficients	Entrez ID	Gene name
VWA3A	0.233	ENSG00000175267	Von Willebrand factor A domain-containing protein 3A
ZBBX	0.226	ENSG00000169064	Zinc Finger B-Box domain containing
PTPRT	0.208	ENSG00000196090	Protein tyrosine phosphatase receptor type T
ASB2	0.201	ENSG00000100628	Ankyrin repeat and SOCS box containing 2
PXYLP1	0.192	ENSG00000155893	2-Phosphoxylose phosphatase 1
STAC2	0.185	ENSG00000141750	SH3 and cysteine rich domain 2
ZNF385D	0.180	ENSG00000151789	Zinc finger protein 385D
SMCO4	0.166	ENSG00000166002	Single-pass membrane protein with coiled-coil domains 4
PTGIS	0.159	ENSG00000124212	Prostaglandin I2 synthase
SATB2	0.156	ENSG00000119042	SATB homeobox 2
HACL1	0.153	ENSG00000131373	2-Hydroxyacyl-CoA lyase 1
CSDC2	0.147	ENSG00000172346	Cold shock domain containing C2
PCNT	0.143	ENSG00000160299	Pericentrin
FOSB	0.140	ENSG00000125740	FosB proto-oncogene, AP-1 transcription factor subunit
PDZD2	–0.137	ENSG00000133401	PDZ domain containing 2
SLC15A3	–0.151	ENSG00000110446	Solute carrier family 15 member 3
ONECUT1	–0.174	ENSG00000169856	One cut homeobox 1
SLC17A6	–0.185	ENSG00000091664	Solute carrier family 17 member 6

## Discussion

In this study, we mainly identified genes preferentially correlated to GMV changes in functional constipation patients by linking gene expression patterns to GMV difference patterns in humans. We found 18 genes’ expression values showing robust correlations with GMV changes in functional constipation, including the orbitofrontal cortex, precentral gyrus, and thalamus. These outcomes could highlight our recognition of the transcriptional features correlated with GMV changes in functional constipation patients.

Here, we found the decreased GMV in the right OFC and its association with constipation scores and depression. As one of the least understood regions, OFC assumes a vital part in emotional regulation, visceral information integration (including sensory and motor information), and decision-making (Kringelbach and Rolls, 2004; Price, 2007; Rolls and Grabenhorst, 2008; Bongers et al., 2009; Rolls, 2019). Additionally, the OFC shows anatomical connection with the cingulate gyrus, amygdala, hypothalamus, and midbrain (Price, 2007), which subsequently portrays its contribution to emotional modulation and visceral coordination. Previous functional MRI (fMRI) studies have reported that the distension of the lower gastrointestinal tract will activate OFC (Derbyshire, 2003), followed by another study that showed that the painful and non-painful gastric stimulation will also activate the right OFC (Guleria et al., 2017). Thereby, these studies further support the vital role of OFC in regulating visceral function. Meanwhile, increased baseline activity in OFC was found in patients with functional constipation and showed a correlation with the sensation of incomplete evacuation (Zhu et al., 2016). Functional constipation is often accompanied by mental issues; the most common are anxiety and depression (Emmanuel et al., 2001; Waters et al., 2013). In this study, we found that the decreased GMV in the right OFC showed association with constipation scores and depression, indicating that the structural injury in OFC might cause the functional abnormality in visceral sensory and motor integration and emotional processing.

In addition, we noticed reduced GMV in the left precentral gyrus and the bilateral thalamus, and the GMV of the bilateral thalamus in patients with functional constipation showed association with PAC-QOL score. The precentral gyrus contributes to controlling the movement execution (Zhu et al., 2016). The structural abnormality of PreG in patients with functional constipation indicated the altered ability to control bowel movement (Hu et al., 2020). Thalamus, as an integrative hub, prominently participates in relaying/integrating/transmitting numerous inputs and connections with various cortical brain areas (Rouiller et al., 1999; Sherman, 2017). Some studies focusing on irritable bowel syndrome revealed the vital role of the thalamus in controlling sensory information (Keszthelyi et al., 2012; Labus et al., 2014) and displayed the activation when distending the rectum (Mayer et al., 2009). Neuroimaging studies depicted numerous functional abnormalities in the thalamus of the functional constipation patients, including the lower amplitude of low-frequency fluctuation in the female functional constipation patients and the decreased nodal degree based on the resting-state fMRI (Jin et al., 2019; Liu et al., 2021). Recently, a study that employed diffusion tensor imaging showed a decreased fractional anisotropy in the fibers communicating with the precentral gyrus, postcentral gyrus, amygdala, and hippocampus in patients with functional constipation, which may imply that the function of integrating the visceral sensory or motor inputs and connecting with other brain regions was impaired in functional constipation patients (Al Omran and Aziz, 2014; Liu et al., 2021).

Currently, we identified that the expression of genes (*VWA3A*, *ZBBX*, *PTPRT*, *ASB2*, *PXYLP1*, *STAC2*, *ZNF385D*, *SMCO4*, *PTGIS*, *SATB2*, *HACL1*, *CSDC2*, *PCNT*, and *FOSB*) showed positive correlations with GMV difference, and the expression of genes (*ONECUT1*, *PDZD2*, *SLC15A3*, and *SLC17A6*) showed negative correlations. For example, there was a positive correlation for *SATB2*, encoding the special AT-rich sequence-binding protein 2, which is a known member of the AT-rich matrix attachment region-binding transcription factor family with a role in the central nervous system and craniofacial development (FitzPatrick et al., 2003; Britanova et al., 2005), also highly expressed and specific for colorectal origins (Magnusson et al., 2011). The more prominent expression of *SATB2* in brain samples with significant GMV decrease in functional constipation may be due to its aberrant expression influence on the brain–gut axis. In contrast, *SLC17A6* acted a negative correlation, i.e., the lower gene expression in brain samples manifested a more notable GMV decrease in functional constipation. *SLC17A6* is a protein-coding gene, also known as VGluT2, highly expressed in glutamatergic neurons. As a primary afferent neurotransmitter, glutamate transfers information from the mucosa to the enteric plexuses and brain. Changes in *SLC17A6* expression could indicate glutamatergic dysfunction in bowel disease (Tong et al., 2001). Even though the genes related to GMV changes in functional constipation is a backhanded technique, we accept that the strategy can suggest valuable discovery on account of such firsthand datasets lacking (Richiardi et al., 2015).

## Limitations

Some limitations should be considered when interpreting our findings. First, we have not collected a larger sample of patients with functional constipation and healthy controls, which restricted the wide application and weakened the statistical robustness. Second, all the functional constipation patients that we enrolled had a history over 1 year, and their medications are different. Some patients only took healthcare products, while some took laxatives occasionally. In this study, we did not take into account the effect of medicines on the results. Third, genes with undetectable expression variation across individuals were omitted in such analysis, since the gene expression data and neuroimage data were acquired from different individuals. Finally, our study is experimental, and further, we should explore whether the identified genes have a causal influence on the altered GMV in functional constipation patients.

## Conclusion

In brief, we performed transcriptional neuroimaging association to define the genes that appear to have correlation with GMV changes in functional constipation. The identified 18 genes, accordantly manifesting prominent relationships between GMV alterations in functional constipation and gene expression value, could be regarded as candidates to detect biological mechanisms of abnormality in functional constipation patients.

## Data Availability Statement

The original contributions presented in the study are included in the article/supplementary material, further inquiries can be directed to the corresponding author/s.

## Ethics Statement

The studies involving human participants were reviewed and approved by the Ethical Committee of Shanghai Tenth People’s Hospital. The patients/participants provided their written informed consent to participate in this study.

## Author Contributions

WC and YZ designed the research, analyzed the data, and wrote the manuscript. WC, LW, and RZ performed the research. WC, LW, RZ, and BY were involved in the clinical assessment. WC, YZ, TH, and JG processed part of the data. GT provided guidance and advice. All authors contributed to the article and approved the submitted version.

## Conflict of Interest

The authors declare that the research was conducted in the absence of any commercial or financial relationships that could be construed as a potential conflict of interest.

## Publisher’s Note

All claims expressed in this article are solely those of the authors and do not necessarily represent those of their affiliated organizations, or those of the publisher, the editors and the reviewers. Any product that may be evaluated in this article, or claim that may be made by its manufacturer, is not guaranteed or endorsed by the publisher.

## Footnotes

1
http://human.brain-map.org
