# Corrigendum: Transcriptomic Signatures Associated With Gray Matter Volume Changes in Patients With Functional Constipation

**DOI:** 10.3389/fnins.2022.878535

**Published:** 2022-03-15

**Authors:** Wangli Cai, Yujing Zhou, Lidi Wan, Ruiling Zhang, Ting Hua, Jian Gong, Bo Yang, Guangyu Tang

**Affiliations:** ^1^Department of Radiology, Shanghai Tenth People's Hospital, School of Clinical Medicine of Nanjing Medical University, Shanghai, China; ^2^Department of Radiology, The First Affiliated Hospital of Dalian Medical University, Dalian, China; ^3^Department of Colorectal and Anal Surgery, Shanghai Tenth People's Hospital, School of Clinical Medicine of Nanjing Medical University, Shanghai, China

**Keywords:** functional constipation, Allen Human Brain Atlas, gene expression analysis, gray matter volume, imaging-genetics

In the published article, there was an error in affiliations 1 and 3. For affiliation 1, instead of “Department of Radiology, School of Clinical Medicine, Shanghai Tenth People's Hospital, Nanjing Medical University, Shanghai, China,” it should be “Department of Radiology, Shanghai Tenth People's Hospital, School of Clinical Medicine of Nanjing Medical University, Shanghai, China.” For affiliation 3, instead of “Department of Colorectal and Anal Surgery, School of Clinical Medicine, Shanghai Tenth People's Hospital, Nanjing Medical University, Shanghai, China,” it should be “Department of Colorectal and Anal Surgery, Shanghai Tenth People's Hospital, School of Clinical Medicine of Nanjing Medical University, Shanghai, China.”

The authors apologize for this error and state that this does not change the scientific conclusions of the article in any way. The original article has been updated.

## Publisher's Note

All claims expressed in this article are solely those of the authors and do not necessarily represent those of their affiliated organizations, or those of the publisher, the editors and the reviewers. Any product that may be evaluated in this article, or claim that may be made by its manufacturer, is not guaranteed or endorsed by the publisher.

